# 3D micropattern force regulates stem cell function

**DOI:** 10.1093/nsr/nwad198

**Published:** 2023-07-17

**Authors:** Antonios G Mikos

**Affiliations:** Department of Bioengineering, Rice University, USA

Biophysical cues of the cellular microenvironment significantly influence cell behaviors by mechanotransduction [1,2]. For example, the mechanical properties of the extracellular matrix modulate human mesenchymal stem cell (MSC) function and guide tissue regeneration [3,4]. Among these mechanical cues, micropatterns with various geometries on 2D substrates have been shown to substantially tune cell behaviors [5]. Considering that stem cells reside in a complex 3D microenvironment *in vivo*, studies on a 2D substrate geometry may not fully recapitulate the critical characteristics of the native 3D stem cell niche [6]. It would be enlightening to understand how mechanosensing of stem cells works in the 3D niche, as it would provide important guidance for the design of the surface topography of tissue-inducing biomaterials. However, it remains unclear how stem cells sense and respond to mechanical signals from a 3D geometry, and, moreover, how a 3D geometry regulates stem cell function and affects tissue regeneration.

**Figure 1.**
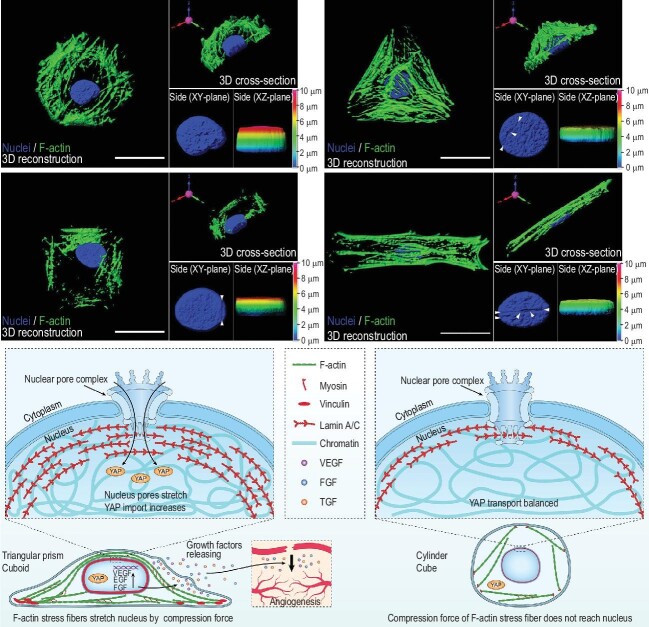
3D micropattern force triggers YAP nuclear entry and modulates stem cells paracrine. In the triangular prism and cuboid micropatterns, the ordered F-actin fibers are distributed over the nucleus, which increased nuclear tension and stretched nuclear pores, thus enhancing the nuclear import of YAP. The activation of YAP enhances the paracrine of MSCs and upregulates the secretion of angiogenic growth factors. Scale bars, 20 µm. Adapted from ref. [7].

Recently, a team led by Professor Shengmin Zhang at Huazhong University of Science and Technology of China published a work in *National Science Review* (Fig. 1) [7], which proposed the concept of a 3D micropattern force and provided the first *in vivo* experimental evidence that the 3D micropattern force regulates stem cell function and promotes tissue regeneration.

Leveraging lithography microfabrication techniques, Professor Shengmin Zhang's team fabricated 3D micropattern arrays with different curvature shapes (cylinder and triangle prism) and aspect ratios (cube and cuboid), and systematically investigated the mechanotransduction of human MSCs triggered by the above 3D micropatterns. Their results demonstrated that the 3D micropattern force could influence the spatial reorganization of the cytoskeleton and cause alteration in the nucleus. Specifically, in the triangular prism and cuboid micropatterns, the ordered F-actin fibers were distributed over the nucleus, which increased the nuclear tension and stretched the nuclear pores, thus enhancing the nuclear import of YES-associated protein (YAP) (Fig. 1). More importantly, the researchers found that the activation of YAP in the triangular prism and cuboid micropatterns enhanced the paracrine of MSCs and upregulated the secretion of angiogenic growth factors. The subsequent skin repair experiment provided the first *in vivo* evidence that enhanced MSCs paracrine by a 3D geometry promoted vascularization and tissue regeneration.

In summary, this transformative research by Professor Shengmin Zhang's team reveals that the 3D micropattern force can regulate stem cell function and promote tissue regeneration by activation of mechanotransduction pathways [7]. Their work is truly groundbreaking as it overcomes the limitation of traditional 2D micropatterns by capturing the characteristics of the natural 3D cell niche and demonstrates the added value of 3D micropatterns to regulate, mechanically, cell function and tissue regeneration. The article is highly meritorious and will be an inspiration to the biomaterials community for innovating a new generation of cell/gene-active biomaterials.
